# Novel mouse line with D277N mutation in the *Plau* gene displays autism spectrum disorder-like traits

**DOI:** 10.3389/fcell.2026.1762737

**Published:** 2026-05-06

**Authors:** Maxim Karagyaur, Olga Averina, Kirill Bozov, Stalik Dzhauari, Anastasia Priymak, Raushana Khaybullina, Oleg Permyakov, Vladimir Popov, Olga Grigorieva, Maria Illarionova, Liliia Shkarina, Mikhail Gulyaev, Dmitry Lebedev, Alexandra Primak, Petr Sergiev, Ekaterina Semina, Polina Klimovich, Larisa Samokhodskaya, Pavel Malkov, Yury Pirogov, Boris Tsygankov, Yuliya Chaika, Vsevolod Tkachuk, Elena Neyfeld

**Affiliations:** 1 Medical Research and Education Institute, Lomonosov Moscow State University, Moscow, Russia; 2 A.N. Belozersky Institute Of Physico-Chemical Biology, Lomonosov Moscow State University, Moscow, Russia; 3 Faculty of Chemistry, Lomonosov Moscow State University, Moscow, Russia; 4 Faculty of Physics, Lomonosov Moscow State University, Moscow, Russia; 5 Scientific Center for Mental Health, Moscow, Russia; 6 Russian University of Medicine of the Ministry of Healthcare of the Russian Federation, Moscow, Russia

**Keywords:** autism spectrum disorder (ASD), cognitive and behavioral tests, CRISPR/Cas9, elevated plus maze, PLAU gene, rs1243306395, three-chamber social test, urokinase-type plasminogen activator (uPA)

## Abstract

**Introduction:**

Genetic technologies provide an opportunity to study the molecular basis of a wide range of hereditary pathologies, including mental disorders. Reproducing of potentially pathogenic genomic variants in cellular and animal models allows establishing their functional significance and possible mechanisms of involvement in the pathogenesis of certain disorders.

**Methods:**

In this study, a genetic variant of urokinase type plasminogen activator (uPA, gene Plau) was modeled in mice using CRISPR/Cas genome editing tool, enabling a better understanding of the role of this molecule and its associated pathways in brain development. The protease uPA plays an important role in the directed migration of neural progenitors, glial, endothelial and immune cells, it participates in axon guidance and maturation of synaptic connections, activation of growth factors and degradation of the extracellular matrix. To study the contribution of the catalytic function of uPA to brain development, we have created for the first time a mouse line carrying the D277N (rs1243306395) mutation. We assessed social activity, anxiety, memory, problem-solving ability and stress resistance of these mice, as well as histological features of their brains.

**Results:**

Timely and correct functioning of the Plau gene ensures adequate positioning of crucial cellular components in the developing nervous system. According to bioinformatic calculations, the D277N (corresponds to the human single nucleotide variant rs1243306395) substitution that happens due to C-to-T mutation in the murine Plau gene may impair the catalytic activity of the uPA protein. While retaining their ability to find solutions in the escape test, this mouse line is characterized by high levels of anxiety, impaired social behavior, slowed learning dynamics (spatial memory), and impaired adaptation to stressors. This behavioral pattern can potentially be interpreted as autism spectrum disorder Histological analysis of the brain and cerebral cortex in Plau-D277N mice revealed brain volume enlargement and cortical thickening of approximately 10-15% compared to wild-type mice.

**Discussion:**

In this study, we draw attention for the first time to the genomic variant rs1243306395 in the Plau gene as a potential cause of autism spectrum disorder and propose the genetically modified Plau-D277N mouse line as a model object for studying the pathogenesis of this disorder. These models can also be used for the development and testing of promising therapeutic approaches and pharmacological agents.

## 1 Introduction

Autism spectrum disorders (ASD) is a group of mental and cognitive developmental disorders characterized by problems with social interaction, and motor and behavioral stereotypes (Hodges et al., 2020; Hirota T. and King B.H., 2023). In some cases, this disease can seriously affect patient’s life, reducing ability to learn, self-care, social integration, family formation and employment. According to previously published data, ASD is diagnosed across all ethnic, racial, and socioeconomic groups, with a prevalence of 0.7%–1% in the general population (Elsabbagh et al., 2012).

Genetic factors and environmental exposures are considered to be the causes of autism spectrum disorders, with genetic predisposition accounting for 64%–91% of all ASD cases. The significant role of genetic factors in the pathogenesis of ASD is confirmed by the increased risk of developing this disease in monozygotic twins (La et al., 2025).

The list of genes potentially involved in the pathogenesis of ASD is constantly being supplemented and expanded. Among them are genes implicated in DNA repair and chromatin packaging (*ASXL3*, *CHD2*, *CHD8*, *DDX53*, *KDM5A/B*, *MECP2*, *POGZ*, etc.), in the formation and functioning of ion channels (*CACNA1H*, *GABRB3*, *GRIN2B*, *KCNQ3*, *KCNQ5*, *SCN2A*, etc.), however, especially numerous are genes participating in brain morphogenesis (*ADNP*, *ANK2*, *ARID1B*, *ASH1L*, *CNTN4*, *CTNND2*, *DSCAM*, *DYRK1A*, *NLGN1/3*, *RELN*, *SHANK2/3*, *SYN1/2*, *SYNGAP1*, etc.). The last ones are responsible for the processes of proliferation and migration of neural progenitors, neurite growth and navigation, synapse formation and maturation, etc. (Apte and Kumar, 2023; Lasser et al., 2023; Daghsni et al., 2018). This correlates with abnormalities in brain structure described in some patients with ASD, such as increased cerebellar and amygdala size, thickening of frontal and temporal cortex, changes in the density and size of cortical microcolumns, changes in cell density of certain brain regions and abnormalities of synapses (Hashem et al., 2020). Taken together, this confirms the importance of proper brain development for the preservation of mental and cognitive function. The brain structure anomalies observed in ASD are quite expected given the genes involved, whose untimely expression or dysfunction can impair the precise positioning of neural progenitor cells in brain tissue and connection into functional neural networks, laying the basis for psychiatric disorders (Jeste, 2015; Primak et al., 2024). Confirmation of their functional significance in cellular and animal models greatly expands the possibilities for their further use for therapeutic and diagnostic purposes in the clinic.

Critical stages of brain development are the migration of neural and glial progenitors, axon growth, and pruning of redundant neural connections - all the stages in which proteases (plasminogen activators, matrix metalloproteinases, etc.) play a key role (Turgeon and Houenou, 1997; Bai and Pfaff, 2011; Primak et al., 2024). One of such key axes involved in the processes of directed cell migration, neurite sprouting and synaptic pruning is the system of plasminogen and its activators, in particular urokinase-type plasminogen activator (uPA) (Krystosek and Seeds, 1981; Shmakova et al., 2021). This molecule, via plasminogen activation, stimulates maturation of matrix metalloproteinases and growth factors, triggers degradation of matrix proteins and repellent molecules, which promotes survival and progression through the matrix of migrating cells, sprouting of growth cones, and removal of nonfunctional neurites and synapses (Krystosek and Seeds, 1981; Mahmood et al., 2018; Shmakova et al., 2021). In addition to its protease activity, uPA also possesses ligand properties. Its partner, the GPI-anchored uPAR receptor, localizes uPA at the leading edge of a migrating cell or sprouting growth cone (Klimovich et al., 2020), and transduces the signal in the cell via co-receptor molecules: integrins, EGFR and a number of GPCRs (Iftikhar et al., 2025). The involvement of the uPA/uPAR signaling axis in the pathogenesis of ASD (Eagleson et al., 2010), and the role of uPAR in neural progenitor migration during brain development (Rahimi-Balaei et al., 2018) have been previously reported. At the same time, the role of urokinase-type plasminogen activator itself, its protease activity and variants within the *Plau* gene in the pathogenesis of ASD have remained unexplored so far.

To investigate the role of uPA protease activity in brain development and psychiatric disease predisposition, we introduced a mutation in its active site (the codon encoding aspartic acid-277 residue). The C- > T substitution (mimicking the genomic variant rs1243306395) caused by cytosine deamination was chosen to introduce this mutation, as it is the most frequent type of mutations found in nature (Ignatov et al., 2017), playing a leading role in the occurrence of hereditary diseases and cancer (Ignatov et al., 2017; Shi and Shen, 2023). The C- > T substitution in the D277 residue leading to a D277N mutation in the active site of murine uPA, according to ProtVar 1.4 and AlphaMissense, is highly likely to be pathogenic, disrupting the catalytic function of the uPA protein (ProtVar, 2025) due to changes in uPA active site conformation and its interaction with substrate. The importance of structural integrity of the uPA active site for the preservation of its catalytic activity is indicated by the high conservation of its amino acid sequence across evolutionarily distant species (from fish (*Danio rerio*) and reptiles (*Naja naja*) to rodents (*Mus musculus*) and humans), despite the fact that the rest part of the uPA molecule undergoes significant changes in amino acid sequence and structure during its evolution. The prevalence of this genomic variant in the human population is extremely rare (0.000085%) (https://gnomad.broadinstitute.org/variant/10-73914122-G-A?dataset=gnomad_r4), which is why its function remained unexplored for a long time. Our study is pioneering in this field.

Modeling this genomic variant in mice, we did not know what phenotypic changes to expect. However, the totality of the results indicates that mice carrying the amino acid substitution D277N (rs1243306395) reproduce behavior patterns characteristic of autism spectrum disorders (Hodges et al., 2020; Hirota T. and King B.H., 2023). The experimental data obtained for the first time convincingly prove the significance of protease activity of uPA protein in brain development and pathogenesis of autism spectrum disorders. The peculiarities of the structure and electrical activity of certain brain regions and their wiring in these animals have yet to be established.

## 2 Materials and methods

### 2.1 Animals

The C57Bl/6J mouse line (Center for Genetic Resources of Laboratory Animals IC&G SB RAS, Novosibirsk, Russia) was used as the parent strain for genomic editing. Mice were kept in individually ventilated cages (IVC system, TECNIPLAST S.p.A., Italy) in specific pathogens-free conditions with free access to pelleted feed and water purified by reverse osmosis, under a light regime of 12/12 (light on at 09:00), in rooms with air exchange rate of at least 15 volumes/h, air temperature 20 °C–24 °C and humidity 30%–70%. Cages containing WT and D227N mice were placed in the same room and maintained under identical conditions: they were housed in adjacent spaces on the rack, received the same food and water, and the group size within each cage was also the same, in order to minimize any potential « cage-related effects » on the animals’ behavior. All animal studies were conducted in accordance with Directive 2010/63/EU (20 October 2010) and were approved by the Local Bioethics Committee of the Research Center “Institute of Mitoengineering of Moscow State University” LLC, (Moscow, Russia) - protocol #79 of 2015. For behavioral and histological studies, we used male mice from the control and experimental groups at the age 3–4 months, with an age difference of no more than 2 weeks between groups.

### 2.2 Creation of a genetically modified mouse line carrying the D277N (rs1243306395) mutation in the *Plau* gene

To create a line of mice carrying the D277N mutation in the *Plau* gene, we used *Streptococcus pyogenes* CRISPR/Cas9 genomic editor (GeneArt™ CRISPR Nuclease mRNA, ThermoFisher Scientific, #A29378) in complex with guide RNA designed using the online resource http://chopchop.cbu.uib.no/: 5′-TCT​GCT​CAC​CAA​TAT​CAT​TA-3’. We used a single-stranded oligodeoxyribonucleotide (ssODN), manufactured by IDT DNA (USA), as a DNA matrix to model the desired genomic variant: 5′-TGGAGCAGCTCATCTTGCACGAATACTACAGGGAAGACAGCCTGGCCTACCATAATAATATTGGTGAGCAGAAAGCTTAGTTACCAGAAAGGCTAAAGTAGTGGTGGGAAA-3’. To synthesize guide RNAs we used HiScribe T7 High Yield RNA Synthesis Kit (NEB, #E2040S) and the DNA matrix obtained using Phanta Max Super-Fidelity DNA Polymerase kit (Vazyme, #P505-d3), primers: 5′-TGTAATACGACTCACTATAGGTCTGCTCACCAATATCATTAGTTTTAGAGCTAGAAATAGCAAG and 5′-AAT​TCA​AAA​AAG​CAC​CGA​CTC, and pX458 plasmid (Addgene, #48138) as a PCR matrix. All procedures were performed according to the manufacturer’s recommendations.

To model the desired genetic variant we prepared an editing mixture containing GeneArt™ CRISPR Nuclease mRNA, guide RNA, and DNA matrix at a final concentration of 25 ng/μL, 12 ng/μL, and 10 ng/μL, respectively. The editing mixture was introduced into single-cell murine embryos by flow microinjection, in oocyte washing medium using two micromanipulators (TransferMan 4R, Eppendorf, Germany) under visual control performed using the ECLIPSE Ti inverted microscope (Nikon, Japan) (Cho et al., 2009). Single-cell embryos were obtained from fertilized female mice stimulated to superovulate by intraperitoneal injection of 40 μl of Inhibin antiserum with foal mare serum gonadotropin (CARD HyperOva® preparation, Cosmobio LTD, Japan, patent No. 5927588) followed by injection of 200 μl (8 EM) of human chorionic gonadotropin 48 h later (hCG, Chorulon® preparation, MSD Animal Health, Merck Corporation, Netherlands) (Averina et al., 2020). A total of 122 of single-cell mouse embryos were treated. 41 embryos developed to the stage of 2 cells (33.6%) were transplanted into the oviducts of pseudo-pregnant surrogate mothers. The resulting offspring was genotyped by Sanger sequencing for the occurrence of the desired D277N mutation in the *Plau* gene using primers: 5′-ACTTTGTGAGACCAGGCTGAC-3′ and 5′-GAGCTGAAATCCAGCCAAGG-3'. Two males and three females identified as carriers of the target heterozygous genomic variant D277N in the *Plau* gene were used for further inbreeding to obtain the desired homozygous population (Plau-D277N) and a population of wild-type mice (not carrying the D277N mutation) for the control group. Some of the pups carried deletions in the target DNA locus and were not used in the study. Whole-genome sequencing of the obtained line was not performed due to the high cost of this method and the limited funding for this study. The contribution of off-target genome editing was reduced by:

1. Utilizing a high-precision gRNA (UCU​GCU​CAC​CAA​UAU​CAU​UA), which has no predicted alternative binding sites in the mouse genome except for the target site in the *Plau* gene (https://crispr.bme.gatech.edu/cgi-bin/crispr/CRISPER_form.cgi?target_database=mm10&tag_type=seq&tag=TCTGCTCACCAATATCATTA&tag_suffix=NGG&CheckBox_no_indel=on&mismatch_no_indel=2&CheckBox_1_del=on&mismatch_1_del=1&CheckBox_1_ins=on&mismatch_1_ins=1&minSeparationUncleavedToCleaved=110&minCleavageProductSizeDifference=0&minAmpliconLength=220&maxAmpliconLength=330&optimalAmpliconLength=275);
2. Using the offspring of homozygous WT and Plau-D277N mice, derived from crosses of heterozygous littermates after genome editing, for all behavioral and other tests.

### 2.3 Assessment of uPA enzymatic activity

To assess the enzymatic activity of uPA in Plau-D277N mice, these and WT mice were anesthetized with a mixture of Zoletil 100 (Virbac) and Xylazal (Biogel) in sterile 0.9% sodium chloride solution (20 mg and 2 mg, respectively, per 1 kg of body weight). The thorax was opened, and blood was collected via cardiac puncture (600–800 μL/mouse), after which the mouse was euthanized by cervical dislocation. 10% (by volume) of 0.218 M sodium citrate was immediately added to the collected blood to prevent clotting. The sodium citrate concentrations recommended for humans (0.109 M) turned out to be insufficient to prevent clotting of murine blood; therefore, the effective anticoagulant concentration of sodium citrate was determined experimentally. The citrated blood was centrifuged at 2000 *g* for 10 min at +4 °C. The resulting blood plasma was aliquoted into polypropylene tubes in 100 μL portions and stored at −80 °C until analysis.

uPA activity was assessed indirectly by measuring plasminogen activation using “Reachrom-Plasminogen” kit (SPA “RENAM,” Russia, #FA-2) - a reagent kit for determining plasminogen activity by the photometric method, in accordance with the manufacturer’s instructions. uPA activity assessment was based on the ability of blood plasma components (predominantly, tPA and uPA) to activate plasminogen and promote the formation of a colored enzymatic reaction product - para-nitroaniline. To evaluate the plasminogen-activating potential of blood plasma samples from Plau-D277N and WT mice the optical density of samples was recorded over time at 0, 30, 60, 105, 150, and 210 min during incubation at +37 °C.

The impact of uPA (rather than tPA or other potential plasminogen activators) in plasminogen activation was confirmed by conducting a similar study, however, in the presence of specific anti-uPA inhibitor BC 11 hydrobromide (Tocris, #4372) at a final concentration of 33 μM. According to the manufacturer’s data, the BC 11 hydrobromide inhibitor is highly specific for the urokinase-type plasminogen activator, but not for other serine proteases.

A comparative analysis of uPA protein levels in the blood plasma of Plau-D277N and WT mice was performed using polyacrylamide gel electrophoresis of plasma samples followed by Western blotting (Burnette, 1981). Samples were normalized by total protein content prior to loading; 50 μg of total protein was loaded into each well. Rabbit Anti-Urokinase antibody [EPR6273] (Abcam, #ab133563) at a dilution of 1:1000 were used as primary antibodies, and goat anti-rabbit immunoglobulin antibodies labeled with horseradish peroxidase (Imtek, Russia, #P-GAR Iss) at a dilution of 1:10,000 were used as secondary antibodies. The signal was detected using Clarity™ Western ECL Substrate (BioRad, #1705060). The signal was registered using the ChemiDoc MP system (BioRad) with equivalent exposure times for the membranes containing samples of Plau-D277N and WT mouse plasma.

### 2.4 Elevated plus maze

The anxiety of mice was assessed in the elevated plus maze (EPM) test (Bailey and Crawley, 2009). A mouse was released into the center of the EPM with its head toward an open arm. The illumination brightness at the maze center was 650–700 lux. The duration of observation was 10 min, during which the following parameters were recorded: the number of visits and the time of staying on the open arm (OA) and closed arm (CA) of the EPM, latent time of entering the OA, the number of peeps out and hangings down from the OA, the number of peeps out from the CA, the number of crosses and the time of staying in the EPM’s center, the number of stands in the center and on the CA of the EPM, the number of defecations, the number of acts and the total time of grooming. Video recording of the experiment was performed.

### 2.5 Three-chamber social test

Possible deviations in the social behavior of mice were evaluated in the three-chamber social test (Yang et al., 2011). Mice have a natural need to approach and explore unfamiliar relatives, which allows us to draw an analogy between the impaired social behavior of mice and the social deficits characteristic of schizophrenia and autism spectrum disorders (ASD) in humans. In the first phase of this test (“Social preference”), the mouse was launched into a central compartment from which it could freely move to the side compartments. An unfamiliar mouse (Stranger #1) was placed in one side compartment in a cylinder with wire walls, and an empty cylinder without a mouse was placed in the opposite side compartment. We assessed the propensity of the mouse to visit or avoid the area around the cylinder with the Stranger #1 mouse and the number of contacts and time spent with the Stranger #1. During the second phase (“Social recognition”), stranger mice were placed in both cylinders: Stranger #1, already familiar to the test mouse from the first phase, and an unfamiliar one (Stranger #2). For the test mouse, we measured the frequency of visits and the total time spent in the areas around the cylinders with the familiar (Stranger #1) and unfamiliar (Stranger #2) mice. All chambers of the setup were uniformly illuminated. Each phase of the test lasted for 10 min. The experiment was videotaped.

### 2.6 Resident–intruder test

The Resident–Intruder test to assess mouse aggressiveness was conducted as previously described (Clipperton and Page, 2023). Briefly, experimental mice (WT and D277N) were housed individually, with no cage changes but under otherwise normal conditions. This isolation allows test mice to establish a territory and increases the likelihood of an attack. Intruder mice (WT) were housed in groups throughout the experiment. Before the test, the intruder was marked with white water-soluble ink. A new intruder was used in each test to reduce the risk of anticipating aggression from the resident mouse, experienced before. To conduct the test, the intruder was placed in the cage with the resident mouse. The mice were then left undisturbed to be videotaped interacting freely for 15 min. During video analysis we assessed the number of contacts initiated by the resident mouse, the number of acts of aggressive grooming by the resident mouse, the number of fights and acts of displaced aggression, as well as the total duration of each of these actions.

### 2.7 Startle reflex

The adaptation of mice to stress was evaluated in the model of “acoustic startle reflex” (Valsamis and Schmid, 2011) by providing a 100 dB sound stimulus of 20 m duration against a background of “white noise” of 60 dB intensity with repetition of the 100 dB stimulus every 15 s. To assess the effectiveness of prepulse inhibition the 100 dB stimulus was preceded by an 80 dB prestimulus for 0.1 s. Each series consisted of ten 100 dB stimuli with or without the prestimulus. The sudden and loud sound stimulus made animals to jump involuntary, and the amplitude of such motor reaction was measured using a vibration detector WTVB02-485 3-axial Vibration Disp + Speed + Amplitude IP68 Waterproof Sensor (Wit Motion, China) and a software package for Windows. To evaluate the effectiveness of prepulse inhibition the motor response to a dual stimulus (80 + 100 dB) was normalized to a motor response to a single stimulus (100 dB).

### 2.8 Extrapolation escape test

To assess the resourcefulness of mice and their ability to find a solution in an unfamiliar stressful situation we used the Extrapolation escape test (RPCOpenScience Ltd, Moscow, Russia) modified for mice (OpenScience, 2025; Kaminskaya, 2025). Water temperature was maintained at +21 °C. The mouse was placed in the water in the cylinder trap and the time of its escape from the trap was recorded. The duration of each experiment did not exceed 180 s. Mice that did not escape within this trial period were assigned the maximum latency of 180 s for analysis. The experiment was videotaped and the following parameters were evaluated: total time to escape, latency before jumping/climbing, latency before diving, duration of the jumping/climbing and diving periods. Each mouse underwent this test only once; we did not train it nor evaluate its learning ability or memory capacities.

### 2.9 Marble burying test

The anxiety of mice and their propensity to repetitive acts were assessed in the marble burying test (Angoa-Pérez et al., 2013). Twenty black-colored glass balls 11 mm in diameter were placed on fresh bedding in the form of a matrix 4 × 5 in a cage glued with black paper from the outside. A mouse was placed in the cage and covered with a transparent (not lattice) lid for half an hour. Afterwards, the number of balls buried more than 2/3 of their diameter was recorded.

### 2.10 Spatial memory test

Trainability and spatial memory of mice were evaluated by their ability to find the “correct” exit from an open chamber with eight arms, each of which is marked with a geometric figure of unique shape. We measured the latent time to find the “correct” exit during the three training attempts, and the test attempts. The training period consisted of the time interval during which the mouse found the “correct” exit (10–30 s). Break periods between each training round (3 rounds in total) lasted 1 h. The mouse was then tested 1 h after the 3rd training round (short-term memory) and 48 h after training (long-term memory). Video recording of the experiment was performed.

### 2.11 Histological analysis of the brain structure

A mouse was sacrificed in a carbon dioxide atmosphere. Its brain was extracted, fixed in 10% formalin solution, and embedded in paraffin. Slices were prepared from paraffin blocks and stained with hematoxylin-eosin. The photos of the stained brain slices were taken using a Leica MZ 9.5 stereomicroscope equipped with a Leica DFC290 camera and Leica Application Suite v3.0.0 software; and a Zeiss Axioskop 40 microscope equipped with an AxioCam MRc5 camera and AxioVersus40AC software v. 4.5.0.0.

### 2.12 Assessment of *Plau* and *Plaur* gene expression in various brain regions of Plau-D277N mice

We hypothesized that the D277N mutation in the *Plau* gene might alter the expression of *Plau* and its associated receptor gene, *Plaur*, in specific brain regions. To test this hypothesis, mice were euthanized by cervical dislocation, their brains were removed, and dissected into the following compartments: the olfactory bulb, hypothalamus, midbrain, thalamus, cerebellum, medulla oblongata, hippocampus, and frontal cortex. Brain tissue samples were homogenized with Qiagen TissueLyser II Bead Mill (Qiagen), and total RNA was extracted using the ExtractRNA kit (Evrogen, Russia, #BC032) according to the manufacturer’s instructions. The obtained RNA was used in single tube reverse transcription and real-time PCR reactions, which were performed using the 5X Genta Single-tube RT-qPCR Master Mix kit, according to the manufacturer’s instructions (GenTerra, Russia, #RT-M-004-XL). Thirty nanograms of total RNA were supplemented into each reaction. Primers and amplification parameters used for the evaluation of expression levels of genes of interest are listed in the Table 1. The dynamic of PCR product amplification was assessed by SYBR Green dye intercalation into the DNA duplex. The procedure of DNA amplification and detection of fluorescence intensity in the FAM channel was performed using the CFX96 Touch Real-Time PCR Detection System, followed by analysis using CFX Manager Software 3.1 (Bio-Rad).

**TABLE 1:** Primers and amplification parameters used for the evaluation of expression levels of target genes.

Primer name	Nucleotide sequence (5’->3′)	Annealing temperature, °C	Amplicon length, bp
mPlau-qPCR-f	GGT​GAA​AAA​CTC​TGA​AGG​TGG​C	56	288
mPlau-qPCR-r	GGT​CTG​TGG​GCA​TTG​TAG​GG		
mPlaur-qPCR-f	AGG​ATG​AGG​ACT​ACA​CCC​GAG	57	288
mPlaur-qPCR-r	TCC​GGT​TTC​CCA​GCA​CAT​CTA​A		
mRplp0-qPCR-f	GAG​AAA​CTG​CTG​CCT​CAC​ATC​CG	59.5	209
mRplp0-qPCR-r	GTG​GTG​ATG​CCC​AAA​GCC​TG		

### 2.13 Statistical analysis

Statistical analysis was performed using SigmaPlot11.0 software (Systat Software, Inc., Erkrath, Germany). The normality of the distribution of numerical data was assessed using the Kolmogorov-Smirnov criterion. The significance of differences between groups was analyzed using Student’s t-test (for pairwise comparisons) or analysis of variance (ANOVA): Newman-Keuls and Sidak-Holm tests for multiple comparisons if the distribution was normal. Analysis of variance (ANOVA) by ranks (Kruskal–Wallis test, Dunn’s criterion) was used to compare groups of data with a non-normal distribution. Data are presented as mean ± standard deviation or median (25%; 75%) depending on the distribution. Differences between groups were considered significant at p < 0.05 for all types of statistical analysis performed.

## 3 Results

### 3.1 Obtaining mice carrying the D277N mutation in the *Plau* gene

**FIGURE 1:**
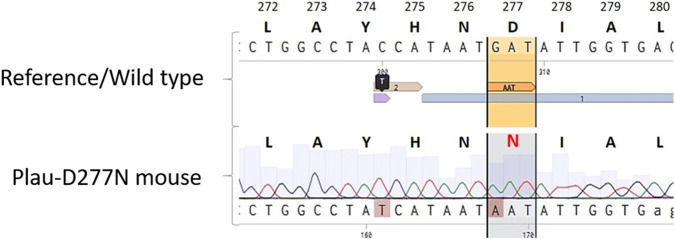
Comparison of amino acid and nucleotide sequences of uPA protein and *Plau* gene, respectively, in wild-type and Plau-D277N mice. The gRNA binding region and its PAM-sequence are shown.

The fact of introducing the desired genomic modification in the *Plau* gene, was confirmed by Sanger sequencing (Figure 1). The body weight of Plau-D277N mice did not differ significantly from that of wild-type mice over the course of the experiment, and at the end point of the experiment (at 5–5.5 months old) was 31 (29.15; 34.5) grams, compared to 31.95 (31.4; 33) grams in wild-type mice n = 8 (Plau-D277N) and n = 11 (WT).

### 3.2 D277N mutation in the *Plau* gene increases catalytic activity of uPA by approximately 40%

**FIGURE 2:**
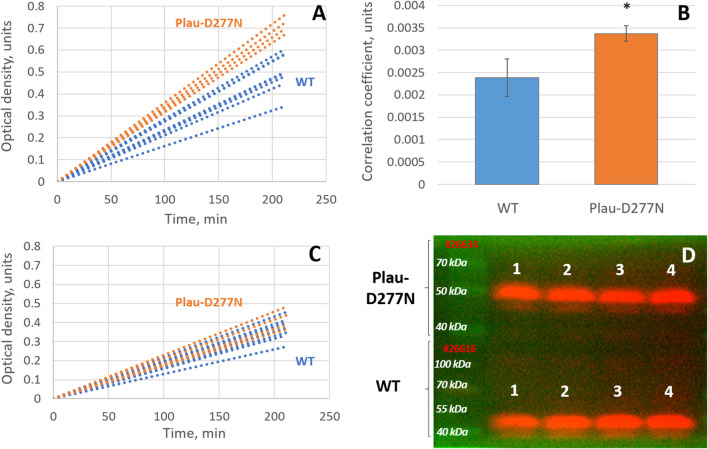
Comparative analysis of the enzymatic activity of uPA from WT and Plau-D277N mice. **(A)** dynamics of the increase in sample optical density due to the accumulation of para-nitroaniline upon addition of blood plasma from Plau-D277N and WT mice; **(B)** Comparison of correlation coefficients for the trend lines of para-nitroaniline accumulation in samples upon addition of blood plasma from Plau-D277N and WT mice, p = 0.001, n = 4 (Plau-D277N) and n = 8 (WT); **(C)** dynamics of para-nitroaniline accumulation upon addition of blood plasma from Plau-D277N and WT mice in the presence of 33 μM BC 11 hydrobromide (a specific inhibitor of uPA enzymatic activity); **(D)** Western blot analysis of uPA in blood plasma from Plau-D277N and WT mice.

An analysis of uPA enzymatic activity in the blood plasma of Plau-D277N and WT mice showed that in Plau-D277N samples a consistently faster increase in optical density was observed due to the accumulation of the product (para-nitroaniline) compared to those containing wild-type mouse blood plasma (Figure 2A). A comparison of the correlation coefficients of the trend lines for the accumulation of para-nitroaniline in individual samples within the groups where blood plasma from Plau-D277N and WT mice was added revealed that blood plasma from Plau-D277N mice exhibits 41% greater activity (promoting faster accumulation of para-nitroaniline) compared to that of wild-type mouse blood plasma: 0.00337 ± 0.00017 vs. 0.00239 ± 0.00042 units, respectively (p = 0.001, n = 4 (Plau-D277N) and n = 8 (WT), Figure 2B).

Blood plasma contains not only urokinase-type plasminogen activator but also tissue-type plasminogen activator (tPA). To determine the significance of uPA specifically in the ability of blood plasma from Plau-D277N and WT mice to activate plasminogen, we performed an inhibition assay—adding the specific uPA inhibitor BC 11 hydrobromide to the reaction. Blocking the enzymatic activity of blood plasma using BC 11 hydrobromide reduces the rate of para-nitroaniline accumulation in samples containing blood plasma from Plau-D277N and WT mice, and also eliminates the difference between these samples. This indicates that the ability of blood plasma to activate plasminogen and initiate the production of para-nitroaniline is largely due to another enzyme (not uPA) - apparently tPA. At the very same time, the disappearance of differences in the rate of para-nitroaniline accumulation between blood plasma samples from Plau-D277N and WT mice as a result of uPA inhibition with BC 11 hydrobromide, indicates that the higher activity of Plau-D277N mouse blood plasma, compared to that of WT mice, is specifically due to the action of uPA (Figure 2C). Western blot analysis of blood plasma samples from Plau-D277N and WT mice revealed no significant differences in uPA content between these samples (Figure 2D). The absence of significant differences in uPA levels in the blood plasma of Plau-D277N and WT mice, coupled with the greater ability of Plau-D277N mouse blood plasma to activate plasminogen, indicates that the D277N missense mutation in the uPA protein enhances its activity by more than 40%.

### 3.3 Behavioral and cognitive features of mice carrying the D277N mutation in the *Plau* gene

**FIGURE 3:**
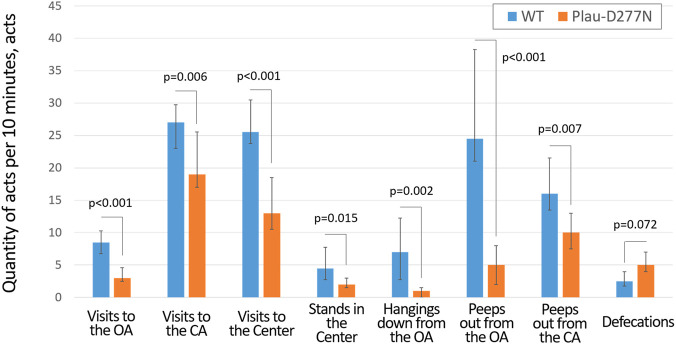
Behavioral features of wild-type and Plau-D277N mice in the elevated plus maze test. WT - wild type, OA- open arm, CA - closed arm, n = 10 (Plau-D277N) and n = 12 (WT).

Examination of the behavior of Plau-D277N line mice revealed a number of features that distinguish them from the cousin wild-type mice line. Thus, Plau-D277N mice revealed significantly more pronounced anxiety in the elevated plus maze (EPM) test, which was expressed in a significantly lower frequency of performing actions requiring courage and decisiveness: walking to the OA and center of the EPM, standing in the EPM center and hanging down from the OA, etc. (Figure 3; Supplementary Figure S1A). Plau-D277N mice spent significantly less time at the OA: 48 (23.7; 72.9) seconds *versus* 91.2 (84.8; 110.7) seconds for wild-type mice (p = 0.004, n = 10 (Plau-D277N) and n = 12 (WT)), and tended to have a higher frequency of defecation acts (p = 0.072, n = 10 (Plau-D277N) and n = 12 (WT)), which are also signs of increased anxiety.

Plau-D277N mice also significantly fewer visited the CA of the EPM: 19 (12.5; 21) acts *versus* 27 (23; 29.75) acts for the cousin wild-type mice (p = 0.006, n = 10 (Plau-D277N) and n = 12 (WT)), which may be explained by their lower frequency of migration between arms. Plau-D277N mice spent most of the EPM test time in the CA: 490.2 (437.1; 596.8) seconds *versus* 456.6 (425.4; 497.3) seconds for the wild-type mice (p = 0.331) for a total test duration of 600 s. This was not a consequence of their physically reduced mobility, but, in combination with other data (reduced number of stands in the center, reduced number of hangings down from the OA, increased frequency of defecations during the EPM test, panic-like behavior in the extrapolation escape task), indicates increased anxiety and caution.

**FIGURE 4:**
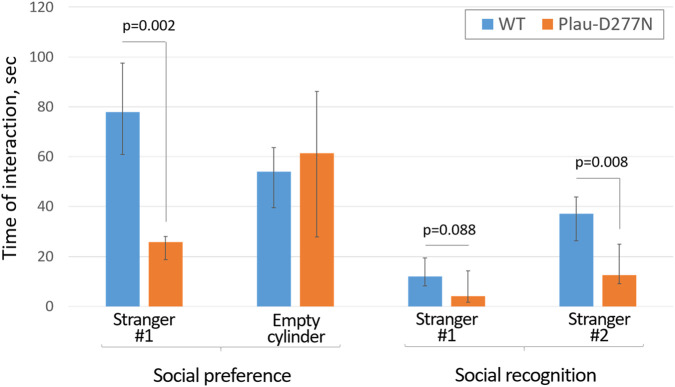
Social behavior features of wild-type and Plau-D277N mice in the three-chamber social test. WT - wild type, n = 10 (Plau-D277N) and n = 7 (WT).

Analysis of the social behavior of Plau-D277N mice revealed a significantly pronounced decrease in their need for intraspecific contacts with both familiar and unfamiliar individuals (Figure 4; Supplementary Figure S1B), which manifested itself in a significant decrease in contact time of a mutant mouse with stranger mice by an average of 3-fold. Interestingly, Plau-D277N mice preferred solitude (empty cylinder chamber) over the company of a new unfamiliar mouse (Stranger #1). Thus, the mean solitude time during the Social preference phase for a Plau-D277N mouse was 61.4 (27.9; 86.25) seconds *versus* 25.8 (18.8; 28) seconds of socializing with the Stranger #1 mouse (p = 0.069, n ≥ 7). In contrast, wild-type reference mice had a predominant need to socialize with the unfamiliar mouse with the mean time of contact during the Social preference phase of 78 (61; 97.5) seconds *versus* 54 (39.5; 63.6) seconds spent alone (empty cylinder chamber).

During the Social recognition phase, wild-type mice spent significantly more time with the new mouse (Stranger #2) than with the Stranger #1: 37.2 (26.4; 43.9) seconds *versus* 12 (8.2; 19.4) seconds (p = 0.008, n = 10 (Plau-D277N) and n = 7 (WT)). For Plau-D277N mice, we also observed a tendency to spend more time with the Stranger #2 mouse than with the Stranger #1 mouse, but such differences were not significant (p = 0.089, n = 10 (Plau-D277N) and n = 7 (WT)). Plau-D277N mice also tended to spend more time in the middle start chamber (with no mice) compared to wild-type mice: 121.4 (75.9; 150.6) seconds *versus* 98.2 (32.9; 143.2) seconds (p = 0.407, n = 10 (Plau-D277N) and n = 7 (WT)).

The totality of the observed behavioral features of Plau-D277N mice in the social test suggests that they have social communication disorders, which is one of the criteria for ASD or schizophrenia.

**FIGURE 5:**
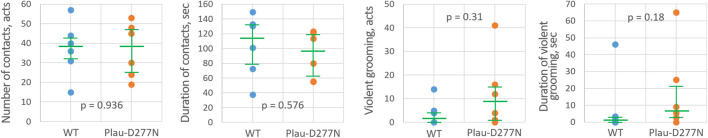
Comparative analysis of some aspects of aggressive behavior of Plau-D277N and WT mice in the resident–intruder test (n = 6 for each group).

The reduced need for social contacts in Plau-D277N mice was accompanied by increased aggression, which was manifested in frequent fights in the cages of Plau-D277N mice and inflicting bites on each other. Under the same housing conditions, wild-type mice did not reveal any increase in conflicts. However, during the resident–intruder test, no significant differences in aggressive behavior between Plau-D277N and WT mice were observed; however, a trend toward increased aggression in Plau-D277N mice was detected across several parameters. For example, Plau-D277N mice showed a tendency toward an increase in the number of episodes and the total duration of aggressive grooming toward intruder mice. The frequency of aggressive grooming episodes in Plau-D277N mice within 15 min of the test was 8 (1.8; 15) *versus* 2 (0; 4.8) in wild-type mice (p = 0.31, n = 6 for each group), and the total duration of aggressive grooming in Plau-D277N mice consisted of 7.2 (3.4; 21.1) seconds *versus* 1.3 (0; 3.1) seconds in WT mice (p = 0.18, n = 6 for each group). Throughout the study, nine fights were recorded, and all of them were initiated by a single mouse from the Plau-D277N group. The total duration of these fights was 27.9 s. No fights were recorded in the WT group. Less pronounced differences were observed for the remaining evaluated parameters (Figure 5; n = 6 for each group).

The tendency to increased aggressiveness could potentially be caused by sensory overload in mice carrying the Plau-D277N mutation, which may be one of the hallmarks of ASD (Hamilton et al., 2011), although this aspect of mouse behavior requires further study.

**FIGURE 6:**
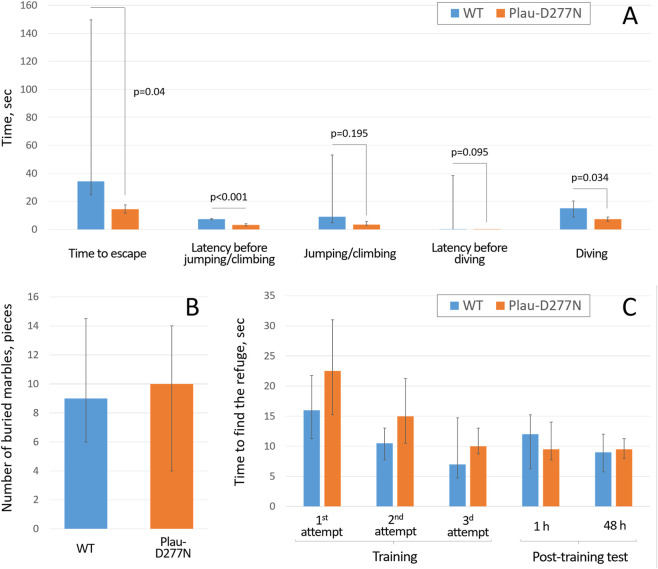
Behavioral features of wild-type and Plau-D277N line mice in the extrapolation escape **(A)** (n = 10 (Plau-D277N) and n = 14 (WT)), marble burying **(B)** (n = 13 (Plau-D277N) and n = 18 (WT)) and spatial memory **(C)** tests. n = 13 (Plau-D277N) and n = 16 (WT).

In the extrapolation escape test, Plau-D277N mice were significantly faster at finding their way out of the trap by guessing to snorkel. They took 14.5 (11.7; 17.9) seconds to solve the task compared to wild-type mice, which took 34.4 (24.8; 149.6) seconds (p = 0.04, n = 10 (Plau-D277N) and n = 14 (WT)) (Figure 6A). Interestingly, 100% of Plau-D277N mice (10 out of 10) were able to solve the task, whereas only 73.3% of wild-type mice (10 out of 14) were able to do so.

Since mice that failed to solve the task within 3 min were assigned the maximum latency of 180 s for analysis, this resulted in a wide distribution of “Time to escape” values. Potentially, the ability of Plau-D277N mice to find their way out of the trap in 100% of cases and to do this more quickly than wild-type mice may be due to advanced logical thinking or a focus on the outcome, which is characteristic of some ASD patients (Blázquez et al., 2025).

If we divide the time spent to solve the extrapolation escape task into individual stages (Latency before jumping/climbing, Jumping/climbing, Latency before diving, Diving), we can see that the overall time reduction in Plau-D277N mice is due to the reduction of each of these stages. Moreover, latency before jumping/climbing and diving time were reduced to the greatest extent in Plau-D277N mice, which, according to the literature (Nimmo-Smith et al., 2020), can be interpreted as an extreme degree of anxiety bordering on panic or an ability to focus on the outcome (Blázquez et al., 2025). Whether the high performance of Plau-D277N mice in solving the escape test is due to panic or an outstanding logical thinking/focus on the result remains to be studied.

The analysis of mice behavior in the marble burying test, which characterizes anxiety and tendency to repeated actions (compulsions) (Angoa-Pérez et al., 2013;; Thomas et al., 2009), did not reveal statistically significant differences between wild-type and the Plau-D277N mice (Figure 6B, (n = 13 (Plau-D277N) and n = 18 (WT))).

Analysis of the trainability and spatial memory of Plau-D277N mice showed that they had a tendency to learn more slowly than wild-type mice, which was manifested by a longer time to find a shelter in each of the three attempts (Figure 6C), although the observed differences were not statistically significant (n = 13 (Plau-D277N) and n = 16 (WT)).

**FIGURE 7:**
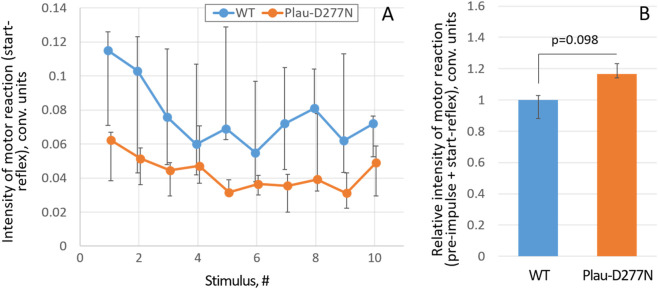
Features of the motor response (startle-reflex) of wild-type and Plau-D277N mice to the sound stimuli without any prestimulus **(A)** or with the prestimulus **(B)**. n = 9 (Plau-D277N) and n = 7 (WT), WT - wild type.

Analysis of the mice motor reaction (startle-reflex) in response to sound stimuli showed that the Plau-D277N mice have a two-fold less intense motor reaction compared to cousin wild-type mice (Figure 7A). Thus, the mean intensity of motor reaction of the Plau-D277N mice in response to the first stimulus was 0.062 (0.038; 0.067) conv. units *versus* 0.115 (0.071; 0.126) conv. units in wild-type mice, although the observed differences are not statistically significant due to the small sample size (p = 0.09, n = 9 (Plau-D277N) and n = 7 (WT)). Decreased motor response to sound stimuli in Plau-D277N mice may be an indication of decreased sensory sensitivity or tunneling of consciousness, which may be the symptoms of ASD. Plau-D277N mice were also characterized by a weak dynamic in mean motor response intensity throughout the study: 0.062 (0.038; 0.067) conv. units for the first sound stimulus *versus* 0.049 (0.029; 0.06) conv. units for the last sound stimulus - a 1.26-fold decrease (p = 0.145, n = 9). At the same time, for the cousin wild-type mice, the degree of reduction in motor response from the first to the 10th sound stimulus was 1.6-fold: 0.115 (0.071; 0.126) conv. units *versus* 0.072 (0.053; 0.077) conv. units (p = 0.097, n = 7). This indicates a tendency for Plau-D277N mice to have a decreased adaptive response to repeated stressors, i.e., persistence in their behavior (which is one of the symptoms of ASD), although the observed differences are not statistically significant.

An additional sign of a decrease in the adaptive response of Plau-D277N mice is a more prominent motor reaction in the modification of startle-reflex test with a sound prestimulus of lower intensity (Figure 7B). Normally, the lower intensity prestimulus should prepare the brain for the main sound stimulus and reduce the motor response to it - the so called, prepulse inhibition. However, in Plau-D277N mice, the mean motor reaction intensity with the prestimulus normalized to the motor reaction intensity without any prestimulus was 1.17 times higher than that in wild-type mice (p = 0.098, n = 9 (Plau-D277N) and n = 7 (WT)), although the observed differences are not significant due to the small sample size.

### 3.4 Morphological and histological features of the brain of mice carrying the D277N mutation in the *Plau* gene

**FIGURE 8:**
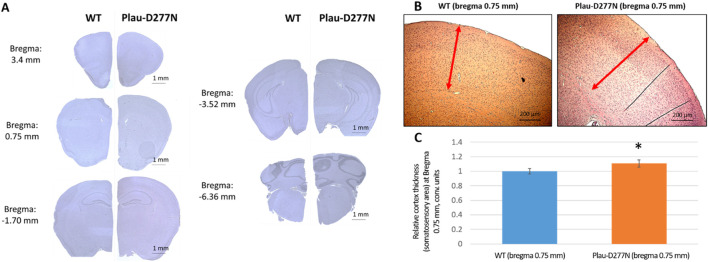
Comparative histologic examination of brain **(A)** and somatosensory cortex **(B,C)** of wild-type and Plau-D277N mice. Staining - hematoxylin/eosin, magnification - 100x. * - p < 0.05, n = 6 (Plau-D277N) and n = 7 (WT). Red arrows indicate cortical thickness on coronal brain slices at bregma 0.75 mm.

Analysis of the brain structure of Plau-D277N mice showed the presence of all brain sections typical of wild-type mice. However, our attention was drawn to the consistently larger brain size of Plau-D277N mice and thicker somatosensory cortex (1.107 ± 0.049-fold) compared to these parameters for cousin wild-type mice (Figure 8; p < 0.05; n = 6 (Plau-D277N) and n = 7 (WT)). A comparative analysis of brain weight in Plau-D277N mice *versus* wild-type mice at the end point of the experiment (at 5–5.5 months old) revealed no statistically significant differences between these groups: 0.5 (0.49, 0.51) and 0.495 (0.49, 0.5) grams, respectively. (p = 0.254, n = 8 (Plau-D277N) and n = 11 (WT)).

### 3.5 Changes in the expression of the Plau and Plaur genes are observed in various brain regions of Plau-D277N mice

**FIGURE 9:**
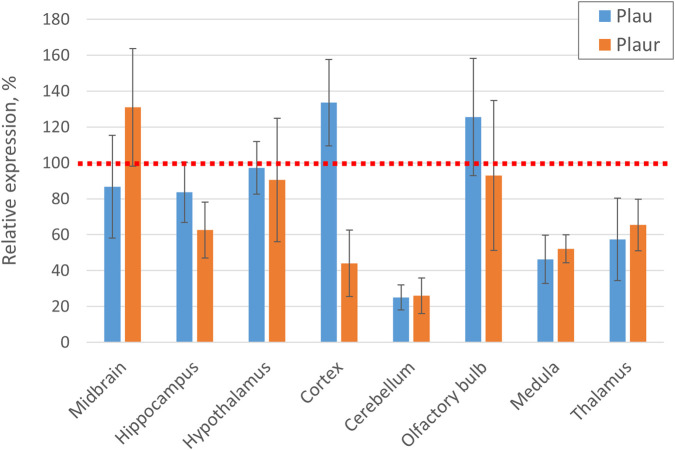
Analysis of *Plau* and *Plaur* gene expression in various brain regions of Plau-D277N mice. The red dotted line indicates the average expression level of Plau or Plaur genes in the respective brain region of a wild-type mouse (n = 7 (Plau-D277N) and n = 6 (WT)).

Analysis of the expression of *Plau* and *Plaur* genes in the brain of Plau-D277N mice revealed that the D277N-mutation in the *Plau* gene itself does not alter significantly expression of these genes. However, reduced expression of *Plau* and *Plaur* was observed in several brain regions (cerebellum, medulla oblongata, thalamus) of Plau-D277N mice (n = 7) compared to wild-type controls (n = 6), Figure 9. The cause and mechanism of this phenomenon remain to be elucidated. We cannot rule it out that this reduction in *Plau* and *Plaur* gene expression may be compensatory in nature against the background of increased catalytic activity of the mutant urokinase-type plasminogen activator.

## 4 Discussion

Genetic variants identified as significantly more frequent in patients with various pathologies in order to obtain the causative and predictive potency require mandatory validation of their functional significance using cellular and/or animal models. Without this, such observations and results are nothing more than correlations (Arévalo et al., 2004; Arévalo et al., 2010; Lin et al., 2013; Karagyaur et al., 2024). This is true for genomic data obtained from psychiatric patients as well. Cellular and animal models allow us to confirm or refute the significance of an identified genetic variant as a causative of a particular pathology and to further understand its role in disease pathogenesis Karagyaur et al., 2022. These models are also convenient objects for testing promising diagnostic and therapeutic methods (Shmakova et al., 2022; Illarionova et al., 2025).

An amazing bonus of using genetically modified animals for modeling hereditary mental disorders is their ability to “disassemble” complex pathologies (e.g., schizophrenia, obsessive-compulsive disorder, autism, etc.) into sets of individual symptoms (i.e., syndromes). This opens up the possibility of establishing the genetic basis and molecular mechanism of pathogenesis of each syndrome. All this makes it possible to consider mental diseases as a set of separate syndromes, each of which may be caused by a certain mutation or malfunction of a specific neural circuit or part of the brain, which allows us to take a new look at the nature of mental diseases, approaches to their diagnosis, classification, and, possibly, therapy. In some cases, even monogenic mutations can produce complex phenotypes due to the gene/protein’s broad involvement in metabolism or intracellular signaling. These pathogenic mechanisms and their particularities can also be dissected in great detail using *in vivo* genetic models.

In this study, we created a line of mice carrying a missense mutation in the catalytic site of the uPA protease (Micecoll, 2025). It was shown earlier that the uPA/uPAR system plays an important role in the development and functioning of brain tissue. Thus, uPA upon binding to its receptor (uPAR), catalyzes the conversion of plasminogen into plasmin and activates signaling pathways that promote cell migration, proliferation, and survival during brain development, and in adult brain regulates the processes of synaptogenesis and the synaptic vesicle cycle (Merino et al., 2020). uPA inhibition attenuates neural cell migration and axonal growth (Semina et al., 2016b), and increased expression of uPA by astrocytic cells from patients with fragile X syndrome has been shown to disrupt activation-induced intracellular Ca2+ influx (Peteri et al., 2021).

Qualitative and quantitative changes in the expressions of uPAR and its ligand uPA have been observed in a variety of epileptic disorders, autism, verbal dyspraxia, perisylvian polymicrogyria, as well as some nonhereditary brain diseases (stroke, brain trauma, multiple sclerosis, Alzheimer’s disease, cerebral malaria, HIV-associated leukoencephalopathy and encephalitis) (Bruneau and Szepetowski, 2011; Semina et al., 2016a; Suleymanova and Karan, 2025).

Despite the obvious role of the uPA/uPAR axis in brain development and functioning, the possible involvement of these molecules in the formation of predisposition to mental disorders is poorly understood. Direct evidence for the role of uPA, its protease activity, and point substitutions in the *Plau* gene in disrupting brain development and laying the basis for psychiatric disorders has not existed to date.

Evaluation of behavioral traits of Plau-D277N mice showed that they had statistically significant higher anxiety and reduced need for intraspecific social interaction. At the trend level, Plau-D277N mice were found to have reduced trainability in the spatial memory test, reduced perception, and impaired adaptation to repeated stressors. At the observation level, these mice had a higher frequency of conflicts and degree of skin damage compared to wild-type mice, indicating the increased aggressiveness in Plau-D277N mice. However, during the resident–intruder test, no significant differences in aggressive behavior between Plau-D277N and WT mice were observed, just the tendency of increased number of episodes and the total duration of aggressive grooming in Plau-D277N resident mice toward intruder mice. Possibly, the lack of the differences observed may be caused by the rather small sample size. We cannot rule out that, with an increased sample size, the differences between Plau-D277N and WT mice in certain aspects of the aggressive behavior may become more pronounced.

Plau-D277N mice visited the central areas and the OA and CA of the EPM less frequently, preferring to spend more time in the CA. This was not a consequence of their physically reduced mobility, but, in combination with other data (reduced number of stands in the center, reduced number of hangings down from the OA, increased frequency of defecations during the EPM test, panic-like behavior in the extrapolation escape task), indicates increased anxiety and caution. Further studies are needed to clarify the type of anxiety and establish the conditions that arise it.

At the same time, Plau-D277N mice retained their ability to solve tasks, and they performed reliably better than cousin wild-type mice, which could potentially be explained by their ability to better focus on some details or simply by a general increased anxiety.

A curious fact turned out to be that long-term maintenance of such mice (for 8 months or more) led to the appearance of symptoms from the digestive system, in particular rectal prolapse. The cause of this clinical manifestation is not known and may be due to both chronic constipation, characteristic of ASD (Al-Beltagi et al., 2023), and weakness of connective tissue, due to impairment of its ability to reorganize due to the *Plau* mutation (Krystosek and Seeds, 1981; Mahmood et al., 2018; Shmakova et al., 2021), or a combination of these factors.

Analysis of the enzymatic activity of mutant uPA with the D277N substitution in its active site revealed that this mutation not only fails to block uPA enzymatic activity but, conversely, enhances it by approximately 40%. This uPA hyperfunction may impair proper positioning of neural progenitors during brain development, promote excessive synaptic pruning, increase blood-brain barrier permeability, and enhance immune cell invasion into tissues, which to some extent may be associated with an increased risk of developing psychiatric and neurodegenerative diseases. Interestingly, increased uPA catalytic activity was accompanied by reduced expression of *Plau* and *Plaur* in several brain regions (cerebellum, cortex, medulla oblongata, thalamus) of Plau-D277N mice compared to wild-type controls. The underlying cause and mechanism of this phenomenon remain unclear. It is possible that this downregulation represents a compensatory response to counteract the elevated catalytic activity of the mutant urokinase-type plasminogen activator.

By modeling this single-nucleotide variant (rs1243306395), we did not expect any specific phenotypic effect because this genetic variant has not been previously described and its functional significance remained unknown (Bey and Jiang, 2014; Cording and Bateup, 2023). At the same time, the whole complex of behavioral features found in Plau-D277N mice, together with the increased brain size and increased cortical thickness, can potentially be interpreted as symptoms of an autism spectrum disorder (Hodges et al., 2020; Hashem et al., 2020; Hirota T. and King B.H., 2023). The phenomenon of histological changes in Plau-D277N mice requires further thorough studies involving animals at different stages of ontogenesis in order to establish the architectural features of the brain in these mice in dynamics.

The only difference between the recorded behavior of Plau-D277N mice and the ASD triad (Hodges et al., 2020; Hirota T. and King B.H., 2023) is their lack of observable stereotypies/compulsions in Plau-D277N mice, which were not detected in the marble burying test. We do not exclude that a propensity for stereotypic behavior could be detected in Plau-D277N mice under native cage conditions during the recording of their habitual daily activity. On the other hand, the propensity for stereotypy in these mice may not be present, as this behavioral disorder may be due to dysfunction of other/additional genes (e.g., *Hoxb8*, *Slc1a1*, *Sapap3*, *Slitrk5*, *Spred2* or *PlxnA3*) (Greer and Capecchi, 2002; Zike et al., 2017; Karagyaur et al., 2025).

To date, there are a number of mouse models of autism spectrum disorders: genetic, environmental, and idiopathic, which fully reproduce the clinical features of ASD and allow to develop the effective therapeutic strategies for the correction and prevention of this disorder (Hamilton et al., 2011; Bey and Jiang, 2014). The main goal of our study was not to create another model of ASD, but to understand the functional significance of proteolytic activity of uPA protein and the role of rs1243306395 single point mutation during brain tissue development. uPA plays a key role in the processes of directed cell migration and nerve fiber sprouting, as well as maturation of neural circuits through activation of plasmin and metalloproteinases, degradation of intercellular matrix, maturation and release of growth factors (Krystosek and Seeds, 1981; Mahmood et al., 2018; Shmakova et al., 2021). The importance of the aspartic acid residue in the active center of uPA is confirmed by its high evolutionary conservatism. Thus, the **D**IAL amino acid pattern in the active center of uPA remains unchanged from fish (*Danio rerio*) and reptiles (*Naja naja*) to rodents (*Mus musculus*) and humans, with the overall degree of identity and homology of uPA amino acid sequences at the level of 40% and 57%, respectively (*Homo sapiens versus D. rerio*) (UniProt.org, 2025A; UniProt.org, 2025B; UniProt.org, 2025C; UniProt.org, 2025D).

Comparison of the behavior of Plau-D277N mice with existing models of autism and clinical symptoms shows a high degree of coincidence of behavioral patterns and features of nervous system development (Daghsni et al., 2018; Hodges et al., 2020; Apte and Kumar, 2023; Lasser et al., 2023; Hirota T. and King B.H., 2023). All of this demonstrates the high importance of appropriate proteolytic activity of uPA protein in the processes of brain tissue morphogenesis, correct positioning of neural progenitors and formation of functional neural circuits within the developing brain.

The high degree of overlap between the symptoms of Plau-D277N mice and existing models of autism suggests the presence in these existing models of some overlapping molecular mechanisms affecting the processes of brain development. We cannot exclude (and this has not been investigated to date) that the *Plau* gene is one of the molecular targets whose expression/function is disrupted by mutations in DNA repair and chromatin packaging genes (*ASXL3*, *CHD2*, *CHD8*, *DDX53*, *KDM5A/B*, *MECP2*, *POGZ*, etc.) or under the influence of epigenetic modifiers (valproic acid, etc.). Further studies of the obtained mouse line are needed, including comparisons with other uPA knock-in and knock-out mouse lines, in order to distinguish between the functions of uPA that depend on its protease activity and those that depend on its interactions with uPA receptor or other partners and are independent of enzymatic activity. The absence of such comparisons is a certain limitation of our study and points to avenues for further research.

The Plau-D277N mouse line we obtained (Micecoll, 2025) still represents a significant enigma. Further studies are required to determine the peculiarities of cellular composition, gene expression, and electrical activity of certain brain regions of such mice, as well as the peculiarities of neural and glial cells positioning, neural connections at the cellular/subcellular level and between the compartments of their brains. We suggest that mutation in the active site of uPA impairs the correct positioning of neural progenitors and the formation of functional neural circuits within the developing brain; however, the reliable cellular and molecular mechanisms of the observed abnormalities in the behavior and brain structure of Plau-D277N mice remain unknown. We also suppose that more information about *Plau* and its role in brain morphogenesis can be obtained while comparing behavior and properties of the declared Plau-D277N murine line with previously described mouse lines with impaired *Plau* protein activity/expression (Finckh et al., 2003; Diaz et al., 2017; Kyyriäinen et al., 2019), however this is the object for further studies.

A separate line of research is to investigate whether the behavioral abnormalities observed in Plau-D277N mice can be corrected by pharmacological approaches and/or intestine microbiota transplantation. On the one hand, these data will allow us to evaluate the efficacy of existing/promising therapeutic approaches for the correction of uPA-dysfunction-caused ASD and, on the other hand, to perform additional pharmacological validation of the observed behavioral phenomena.

An interesting continuation of this work could be the study of such mice in adulthood or after the chronic stress exposure, which will potentially allow to further exacerbate the behavioral abnormalities observed in them, if the assumption is correct that chronic stress and aging of the organism provoke the progression of mental disorders. The aged Plau-D277N mice could potentially also be a genetic model for Alzheimer’s disease (Diaz et al., 2020), but this remains to be established.

The prominent clinical and histologic pattern exhibited in mice carrying the single point mutation rs1243306395 within the *Plau* gene supported previously published data of high significance of the uPA/Plau molecule in brain development. Modeling of the rs1243306395 genetic variant enhanced the catalytic activity of the uPA protein rather than causing its knockout, suggesting that not only the inactivation of uPA but also changes in its activity, and possibly its specificity, may disrupt the highly coordinated process of brain morphogenesis. If these findings are confirmed in other animal species, more attention should be attracted to such SNVs as potential cause of psychiatric disorders, including autism spectrum disorders.

## Data Availability

The original contributions presented in the study are included in the article/Supplementary Material, further inquiries can be directed to the corresponding authors.
