# Supplementary figures and images for: Novel mouse line with D277N mutation in the Plau gene displays autism spectrum disorder-like traits
Source: Front Cell Dev Biol. 2026 May 6;14:1762737. doi: 10.3389/fcell.2026.1762737 (PMC13187116; doi:10.3389/fcell.2026.1762737)

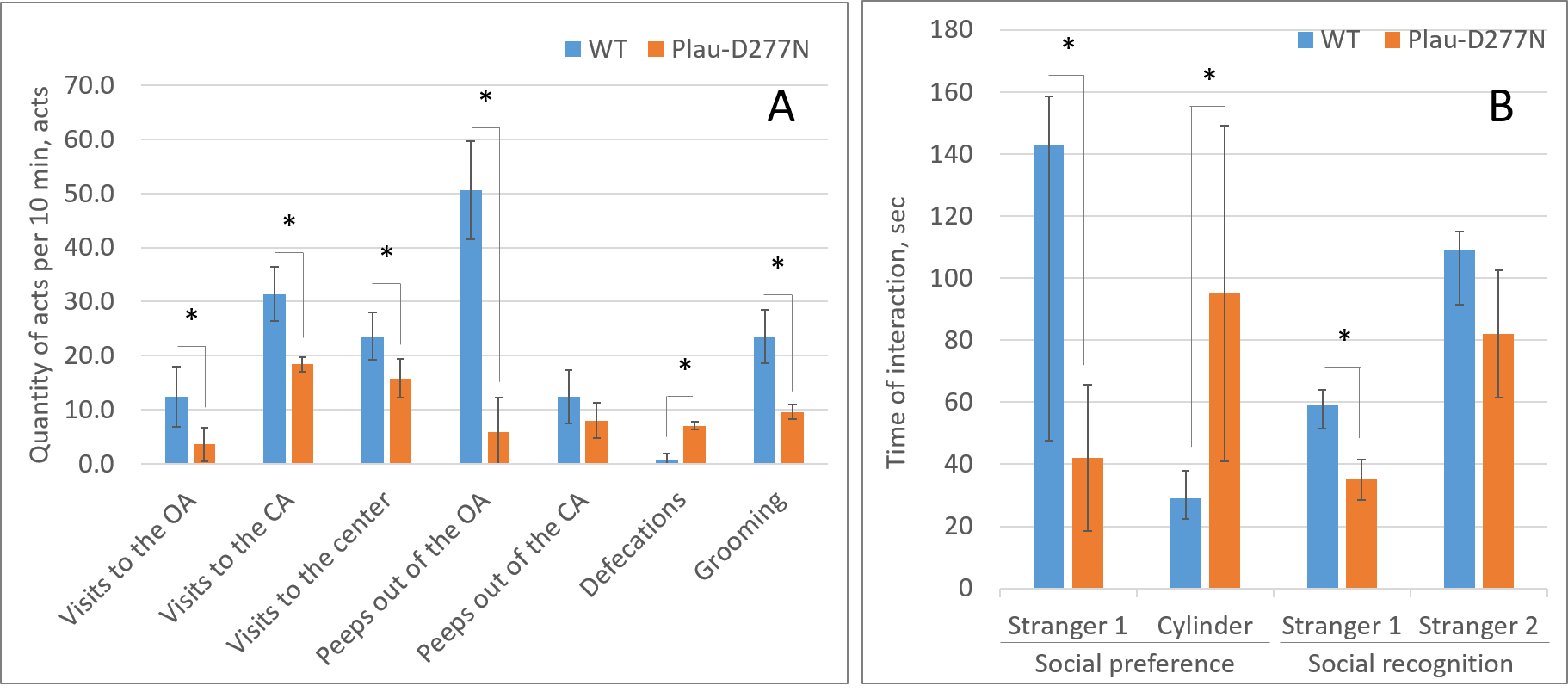

Supplement: Supplementary file 1 [file Image1.tif]
